# The improvement in neurocognitive functioning in anorexia nervosa adolescents throughout the integrative model of psychotherapy including cognitive remediation therapy

**DOI:** 10.1186/s12888-018-1984-4

**Published:** 2019-01-09

**Authors:** K. Kucharska, D. Kulakowska, M. Starzomska, F. Rybakowski, K. Biernacka

**Affiliations:** 1 The Specialist Eating Disorder Unit, Institute of Psychiatry and Neurology, Warsaw, Poland; 2 Institute of Psychology, Faculty of Christian Philosophy, Cardinal Stefan Wyszynski University in Warsaw, Warsaw, Poland; 3 The Department of Adult Psychiatry, Poznan University of Medical Sciences, Poznan, Poland; 4 The Department of Child and Adolescent Psychiatry, Institute of Psychiatry and Neurology, 9 Sobieski, 02-957 Warsaw, Poland

**Keywords:** Neurocognition, Anorexia nervosa, Psychotherapy, Cognitive remediation therapy

## Abstract

**Background:**

Patients with anorexia nervosa (AN) experience difficulties in neurocognitive functioning in the acute phase of illness which might be related to clinical presentation, but also in the apparently remitted state after weight recovery. Among the most commonly reported persistent deficits is cognitive inflexibility, which can be interpreted as a vulnerability trait or a “neuropsychological scar” reflecting the detrimental effect of prolonged semi-starvation in patients with a long duration of illness. Studies of adolescent samples with a relatively short clinical course may enable avoiding the effect of prolonged illness and help to determine whether neuropsychological deficits are trait or state dependent.

The aim of this study is to assess cognitive functioning in adolescents with AN before and after the inpatient treatment programme, including cognitive remediation therapy (CRT).

**Methods:**

Forty-seven adolescent female inpatients with AN diagnosed according to DSM-5 and fifty healthy female adolescents matched for the education level and age were recruited. The patients underwent a multimodal treatment including a ten-week CRT. The standardized and cross-validated neuropsychological (Trail Making Test – TMT A and B, Color-Word Stroop Task – CWST, Ruff Figural Fluency Test – RFFT) and clinical measurements (Beck Depression Inventory – BDI, Eating Attitude Test – EAT-26, Yale-Brown Obsessive Compulsive Scale – Y-BOCS) were used to assess both clinical (in the acute phase and after partial weight recovery) and control subjects.

**Results:**

Initially, AN patients performed significantly worse compared to the controls, but afterwards, inpatient treatment improvement was noted on all examined measures. In a few subtests (TMT, CWST) performance of AN patients after the programme was still significantly poorer than in HC.

**Conclusions:**

Cognitive inflexibility in adolescent AN patients, as measured with TMT, CWST, and RFFT tends to improve after therapy. Nevertheless, a few neuropsychological subtests which did not show complete normalization may warrant attention in subsequent studies. Further research including control intervention is needed to conclude whether CRT intervention affects the outcome.

**Electronic supplementary material:**

The online version of this article (10.1186/s12888-018-1984-4) contains supplementary material, which is available to authorized users.

## Background

There is a substantial body of research evidencing impaired neurocognitive functioning amongst patients with feeding and eating disorders [1] anorexia nervosa (AN), in particular asa consequence of the illness per se [2, 3] or underlying traits for AN to develop [4–10]. Neurocognition consists of the following main processes: memory, attention, visuospatial functions, and broadly understood executive functions [11].

A growing body of research shows that neurocognitive impairments in eating disorders are primarily in relation to executive functioning and visual-spatial functioning [12–17]. Two aspects of executive functioning have been described as possible endophenotypes for AN, namely: weak central coherence (i.e. a bias towards detail accompanied by a limited ability to understand context or to “see the big picture” (e.g. Happé & Frith 2006) [18], and set-shifting difficulties or cognitive rigidity (i.e. the inability to move back and forth between different tasks or mental sets in response to changing goals or environmental experiences) [10, 19].

Systematic reviews have shown that neurocognitive deficits were present in samples with eating disorders in contrast to healthy control groups [20–27]. There may have been predisposing trait factors for developing AN which stay in line with the neurodevelopmental model of the illness. It is also possible that they may have been intensified with weight loss and duration of the illness [11, 28] and they may have persisted after recovery [16, 17, 29–31].

Some studies have concluded that neurocognitive deficits can result from psychological variables such as a preoccupation with food and body image [32]. Other reports have found differences in brain structure and function between patients with eating disorders and healthy control groups [2, 33]. Both the hippocampi and anterior cingulate show a reduced thickness of grey matter in Anorexia nervosa, therefore neuropsychological processes associated with these structures, such as attentional switching, set-shifting, working memory, and spatial reasoning are commonly impaired in AN patients. In addition, research has demonstrated that impairments of neurocognitive performance, namely behaviour pattern switching disorders, were present among mothers of children with AN, AN patients, and their siblings which were consistent with neurobiological models [5, 17, 34].

There is a growing body of evidence mainly based upon single-case studies or case series on cognitive remediation therapy in eating disorders, including four randomized controlled trials [35–37] supporting the efficacy of such intervention in adults in improving set-shifting and quality of life, reducing attrition, and ED symptoms [38]. Similarly, a number of studies have evidenced the beneficial effect of CRTs (Cognitive Remediation Therapy) on neurocognitive functioning in children and adolescents [39–42] with no RCT published so far in the younger group [38].

The aims of this study were: 1) to assess post-therapy changes in neurocognitive functioning of patients with AN after completion of the inpatient programme, including Cognitive Remediation Therapy (CRT), to find out whether these impairments are present only in the acute phase of the illness or persist after weight improvement; 2) to compare neurocognitive functioning of patients with AN and healthy controls (HC); 3) to determine the relation of such deficits to patients’ age, education, body mass index (BMI), level of depression, the mean score of obsessive-compulsive symptoms scale, and the severity of core eating disorder psychopathology. Consequently, the aim of the current study was to evaluate patients’ perspective and satisfaction level of CRT (Cognitive Remediation Therapy).

## Methods

A total of ninety-seven female participants, including forty-seven adolescents aged 16–18 years (mean age = 16.46 years, SD = 1.21) diagnosed with AN were recruited from The Department of Child and Adolescent Psychiatry in The Institute of Psychiatry and Neurology in Warsaw (Table 1). They were assessed after giving informed consent. Approval for the study was obtained from the ethics committee of the Institute of Psychiatry and Neurology in Warsaw, Poland (reference number 7/2015 KB IPiN). Informed consent of participation in the study was obtained from all participants (or their parent or legal guardian in the case of children under 16). Patients with a diagnosis of anorexia nervosa (DSM-4 or DSM-5307.1) and BMI ≤ 10th age-percentile at the beginning of the inpatient treatment were included. All participants were right-handed. A vast majority of the patient group was diagnosed with either restrictive AN (*n* = 39;82.9%) or AN binge-purge (*n* = 8;17.0%). The clinical inpatients were consecutively recruited in the years 2012–2015. All were primary or secondary care referrals to a specialist unit for eating disorders. Seven patients were on antidepressants with no effect on the QTc interval (3 on sertraline 100–150 mg/d) and (4 on fluoxetine 20–40 mg/d) due to a concurrent depressive episode. The mean age of onset was 13.99 years (SD = 1.64).

**Table 1:** Demographic and clinical data in three subject groups

Variable	AN 1 (*n* = 47)	AN 2 (*n* = 47)	HC (*n* = 50)
Age	16.46 (1.21)	16.46(1.21)	16.62(1.07)
Age of illness onset	13.99(1.64)	13.99(1.64)	–
Height (cm)	165.66(6.70)	165.66(6.70)	166.60(5.32)
Weight (kg)	40.99(5.09) ^### ***^	47.26(4.82) ^###^	62.41(6.00)
BMI percentile	2.38 (5.45)	11.31(11.02)	67.34(15.82)
BMI	14.84(1.61) ^### ***^	17.07(1.09) ^###^	22.55(1.67)
Years of Education	10.53(1.18)	10.53(1.18)	10.62(1.07)
BDI	23.34(7.32) ^### ***^	9.62(8.99)	10.00(1.06)
EAT-26	32.42(17.73)^### ***^	17.49(17.48)^###^	8.80(6.10)
Y-BOCS Obsessivethoughts	9.49(4.93) ^### ***^	4.96 (4.42) ^###^	4.28(3.86)
Y-BOCS Compulsive Behaviours	7.42 (5.16) ^### ***^	3.87(3.79) ^###^	3.10(3.26)

*BMI* Body Mass Index, *EAT-26* Eating Attitude Test-26, *BDI* Beck Depression Inventory, *Y-BOCS* Yale-Brown Obsessive Compulsive Scale

*AN1* Patients with anorexia nervosa before therapy, *AN2* Patients with anorexia nervosa after therapy, *HC* Healthy controls

Mean and standard deviation presented

^*^p ≤ 05 versus AN2, ^**^p ≤ 0.01 versus AN2, ^***^p ≤ 0.001 versus AN2, ^#^ *p* ≤ 0.05 versus HC, ^##^ *p* ≤ 0,01 versus HC, ^###^ *p* ≤ 0.001 versus HC

A comparison group of fifty non-clinical healthy subjects (mean age = 16.62 years, SD = 1.07), matched for age and education with a normal body weight were recruited in local schools. The exclusion criteria for both subject groups included habitual drug or alcohol abuse, secondary neurologic disorders (e.g. epilepsy or dementia), pregnancy, and other psychiatric diagnoses. Furthermore, healthy participants were excluded if they had symptoms of eating disorders, menstrual irregularity, a family history of an eating disorder or a body mass index (BMI = weight in kilograms divided by height in meters squared) lower than 18 and greater than 26 or a weight outside the 10th or 90th percentile. The weight and height were measured at the time of diagnosis and the BMI was calculated for each patient and plotted on the relevant WHO charts.

Healthy participants were examined once while patients were assessed twice*:* before therapeutic intervention at the beginning of the second week after admission and after therapy prior to discharge. Forty**-**seven patients out of fifty initially recruited participants successfully completed therapy and underwent evaluation twice. One participant did not complete post-intervention outcome measures and two patients discharged themselves prematurely against medical advice.

Various neurocognitive functions were measured using well-known tests/batteries:

- Trail Making Test (TMT, part A and B) – a test of visual attention and attentional switching; TMT is part of Halstead-Reitan neuropsychological battery of tests used in the clinical diagnoses of brain disorders. The test is used for evaluating visual perception, visual-motor coordination, and visual-spatial operational memory. The test consists of parts A and B, and involves matching circles with numbers and letters. Part A of TMT measures psychomotor speed and visual-motor coordination. Part B of TMT, in addition to the skills tested in part A, also serves to evaluate visual-spatial operated memory and executive functions.
- Color – Word Stroop Task (CWST) – a test of attention, executive functioning; Tests used to measure executive functions by using the interference effect were developed by Stroop in 1935. The first part of the test involves quickly reading the names of colours written in black on a sheet of paper and measuring the speed at which it is accomplished. In the second part, the test taker is given a sheet of paper with the names of colours written in red, yellow, blue, and black. The result of the test is calculated as the time required for naming the colours and the number of errors. The test also measures the interference between the meaning of the word and the colour, as well as the time differential and the error differential in parts two and one, as ignoring one factor reduces the speed of the task. The test is used to examine frontal lobe functions, cognitive functions, attention span, mental flexibility, executive functions, and operational memory. The Stroop Test has been used to diagnose cognitive functions in people with mental disorders such as obsessive-compulsive disorder and affective disorders.
- Ruff Figural Fluency Test (RFFT) – a test of nonverbal capacity for fluid and divergent thinking, ability to shift cognitive set, planning strategies, and executive ability to coordinate this process.

Additionally, three clinical measures were used:

- Eating Attitudes Test (EAT-26) – a standardized, self-report measure of symptoms and concerns characteristic of eating disorders. The tests are rated on a six-point scale (always, usually, often, rarely, sometimes, and never) in response to how often the individual engages in specific behaviours.
- The Beck Depression Inventory (BDI); an instrument for the self-rating of symptoms of depression**.**
- Yale-Brown Obsessive Compulsive Scale (Y-BOCS) allowing evaluation of OCD traits or symptoms (Table 1).

### Therapeutic inpatient programme

The patient group underwent the twelve-week integrative model of therapy that integrated elements from systemic family therapy, group therapy, art therapy, psychoeducation, individual therapy, and CBT-based Social Cognitive training in conjunction with cognitive remediation therapy to achieve a more efficacious outcome.

The Cognitive Remediation Therapy – ED module directly addresses the specificity of cognitive style and neurocognitive deficits of AN patients.

### Cognitive remediation therapy (CRT)

The aim of CRT is to stimulate cognitive functions through various modules of CRT (Cognitive Remediation Therapy) resource pack for children and adolescents with AN [43–45] delivered face to face with some specific tasks chosen from Cogpack software (version 8.6). CRT is delivered by a trained psychologist once a week for 60 min in a ten-week programme (10 sessions) in a hospital ward setting. All sessions include the following structure such as: psychoeducation, practical exercises, reflection, and discussion alongside homework tasks. CRT (Cognitive Remediation Therapy) focuses on deficits in processing information and improving cognitive strategies. It allows participants to see their own strengths and weaknesses regarding attention (selective, divided, alternating attention), memory (especially short-term memory), visual and spatial functions, abstract and holistic thinking, and executive functions such as planning, cognitive flexibility, and problem solving. There are five modules of CRT which are aimed at improving attention function, as well as visual and spatial skills; improving memory; improving executive functions; developing abstract and holistic thinking; and increasing cognitive flexibility. Each module consists of two sessions focusing on a given subject and the kind of activities which enhance a given variable. CRT (Cognitive Remediation Therapy) allows therapists a certain level of flexibility in matching suitable tasks depending on the ability level of participants and their motivation to participate in the training. After completing the training, patients were asked to fill in a self-descriptive survey consisting of evaluation questions about the therapist’s work and their own work, as well as the benefits which, in their opinions, they gained after the training.

Twenty-five adolescents who participated in the study have completed CRT (Cognitive Remediation Therapy) satisfaction questionnaires after provision of treatment. The evaluative survey comprised a part which evaluated classes about developing skills of social cognition and neurocognition. The participants answered 12 close-ended questions which evaluated the training on a three-stage scale and two open-ended questions about the benefits gained.

Statistical analyses were performed using Statistica software for Microsoft Windows. After the data of the 97 subjects were collected, the Smirnov-Kolmogorov test was used to analyze data distribution. The T-test for dependent and independent samples and the Wilcoxon test or U-Mann Whitney test (for distribution different than normal) were used.

Correlations were analyzed by Pearson linear correlation or the Spearman test.

## Results

### Demographic and clinical data

**Fig. 1:**
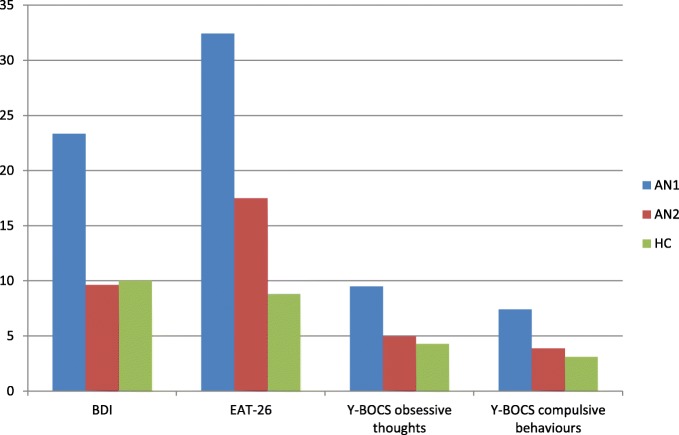
Clinical data for three subject groups. BDI - Beck Depression Inventory, EAT-26 - Eating Attitude Test-26, Y-BOCS - Yale–Brown Obsessive Compulsive Scale. AN1 - patients with anorexia nervosa before therapy, AN2-– patients with anorexia nervosa after therapy, HC - healthy controls

Demographic and clinical data for the three subject groups: patients with anorexia nervosa before (AN1) and after therapy (AN2), as well as healthy controls (HC), were reported in Table 1 and Fig. 1.

No statistically significant differences were found in age and education between AN groups and HC. There was a significant post-therapy difference between patient groups in weight (t(46) = − 10.94; *p* ≤ 0.001) with higher scores in AN patients after therapy. Statistically significant differences were found in weight between AN patients before therapy (AN1) and HC (t(95) = − 18.88; *p* ≤ 0.001), as well as between AN patients after treatment (AN2) and HC (t(95) = − 13.64; *p* ≤ 0.001). Statistically significant differences were found between AN1 and AN2 in the mean BMI score (t(46) = − 10.96; *p* ≤ 0.001) with higher scores in AN2. Results showed statistically significant differences in BMI between AN1 and HC (t(95) = − 23.16; *p* ≤ 0.001) and between AN2 and HC (t(95) = − 18.87; *p* ≤ 0.001) with higher scores in HC.

T-tests revealed a significant difference between AN1 and AN2 in the mean EAT-26 score (t(46) = 6.57; *p* ≤ 0.001) with lower scores in AN2. Statistically significant differences were found in the mean EAT-26 score between AN1 and HC (t(95) = 8.88; *p* ≤ 0.001), as well as between AN2 and HC (t(95) = 3.31; *p* ≤ 0.001) with lower scores in HC.

Statistically significant differences were found between AN1 and AN2 in the mean BDI score (t(46) = 9.21; *p* ≤ 0.001) with lower scores in AN patients after therapy. Results also showed statistically significant differences in BDI between AN1 and HC (t(95) = 10.87; *p* ≤ 0.001) with lower scores in HC. No statistically significant differences were found in the mean BDI score between AN2 and HC.

Statistically significant differences were found between AN1 and AN2 in Y-BOCS (obsessive thoughts) (t(46) = 6.89; *p* ≤ 0.001) with lower scores in AN after therapy. There were statistically significant differences in this scale between AN1 and HC (t(95) = 5.81; *p* ≤ 0.001) with lower scores in HC, but no statistically significant differences were found in obsessive thoughts between AN2 and HC.

There were found statistically significant differences between AN1 and AN2 in the mean Y-BOCS subscale of compulsive behaviours (t(46) = 5.78; *p* ≤ 0.001) with lower scores in AN after treatment. There were also statistically significant differences in the mean score of the scale between AN1 and HC (z = 3.92; *p* ≤ 0.001) with lower scores in HC**;** however, no statistically significant differences were found between AN2 and HC.

### Neurocognitive measures

#### Trail making test (TMT)

Statistically significant differences were found between AN1 and HC (t(95) = 3.64; *p* ≤ 0.001) and between AN2 and HC (t(95) = 2.28; *p* ≤ 0.05) in TMT part A. Statistically significant differences were also found in TMT part B between AN1 and AN2 (t(46) = 3.78; *p* ≤ 0.001) and between AN1 and HC (t(95) = 5.15; *p* ≤ 0.001).

#### Color – Word Stroop task (CWST)

Patients with AN before therapy were found to have longer latencies for reading the names of colours in black as compared to AN2 patients (t(46) = 5.59; *p* ≤ 0.01). Again, patients with AN (AN1 and AN2) demonstrated significantly longer latencies than HC (t(95) = 5.59; *p* ≤ 0.001; z = 5.29, *p* ≤ 0.001, respectively). The analyses revealed that AN1 patients demonstrated significantly longer latencies for naming ink colours of incongruent words compared to those patients after therapy (t(46) = 2.42; *p* ≤ 0.05). It appeared that AN1 patients showed longer latencies in this task than HC (t(95) = 3.45; *p* ≤ 0.001). Additionally, analyses showed statistically significant differences between patients with AN (both AN1 and AN2) and HC in error rates for naming ink colours of incongruent words (z = − 2.58; *p* ≤ 0.01; z = − 2.54; *p* ≤ 0.01), with more errors in patients.

#### Ruff figural fluency test (RFFT)

Performance on RRFT (in total) in AN patients throughout the therapy showed marked improvement (t(46) = − 8.88; *p* ≤ 0.001). Statistically significant differences were also reported between the AN1 group and HC (t(95) = − 3.4; *p* ≤ 0.001) for RRFT, with better performance for patients after therapy. The error ratio in RFFT for both patient groups was higher than for HC (t(95) = 2.05; *p* ≤ 0.05; t(95) = − 2.04; *p* ≤ 0.05, respectively). Statistically significant differences were found between patient groups for rotation that was higher in patients after treatment (z = 2.12; *p* ≤ 0.05). There were also statistically significant differences between AN1 patients and HC, as well as between AN2 patients and HC (z = − 2.20; *p* ≤ 0.05), with better performance in HC.

**Fig. 2:**
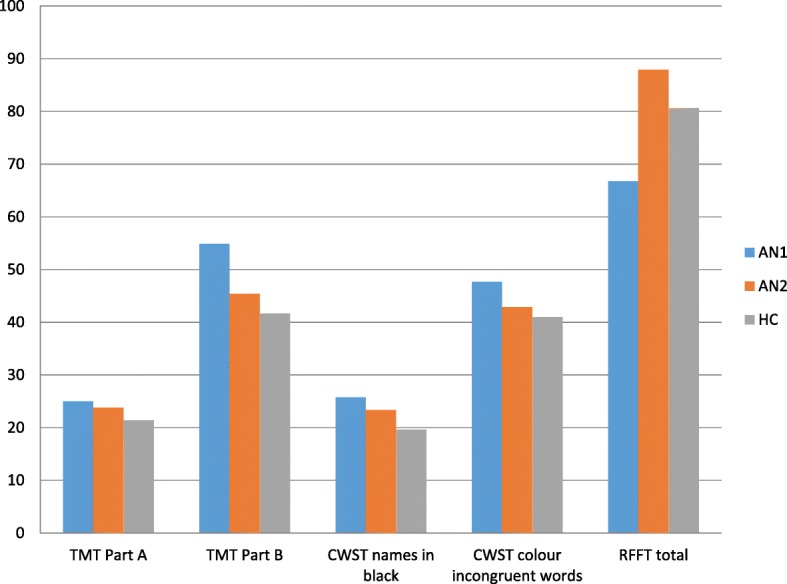
Neurocognitive test results in three subject groups. TMT - Trail Making Test, CWST - Color-Word Stroop Task, RFFT - Ruff Figural Fluency Test. AN1 - patients with anorexia nervosa before therapy, AN2 - patients with anorexia nervosa after therapy, HC– healthy controls

Neurocognitive test results of patients with anorexia nervosa before and after neurocognitive training and healthy controls (HC) are reported in Table 2 (Fig. 2).

**Table 2:** Neurocognitive test results in three subject groups

	AN 1 (*n* = 47)	AN 2 (*n* = 47)	HC (*n* = 50)
Trail Making Test (TMT)			
Part A (s)	25.00 (5.23) ^###^	23.81 (5.89) ^#^	21.38 (4.55)
Part B (s)	54.89 (16.26) ^###***^	45.36 (11.56)	41.68 (7.78)
Part A + Part B	79.89 (18.7) ^###***^	69.17 (13.79) ^#^	63.06 (10.65)
Color - Word Stroop Task (CWST)			
Reading colour names in black - RT (ms)	25.74 (5.41) ^###**^	23.34 (4.54) ^###^	19.64 (5.32)
Reading colour names in black - error rates	0.02 (0.14)	0.11 (0.48)	0.00 (0.00)
Naming colour of word-different - RT (ms)	47.66 (10,85) ^###*^	42.89 (13,16)	40.98 (8,08)
Naming colour of word-different - error rates	0.47 (0.93) ^##^	0.47 (0.93) ^##^	1.16 (1.27)
Ruff Figural Fluency Test (RFFT)			
Ruff Figural Fluency Test – total	66.74 (23.95) ^###***^	87.91 (25.18)	80.64 (15.65)
The Error Ratio	0.55 (1.14) ^#^	0.34 (0.38) ^#^	0.22 (0.17)
Rotation	0.06 (0,25) ^#*^	0.40 (0.97)	0.54 (1.15)
Enumeration	0.15 (0.51)	0.11 (0.48)	0.12 (0.33)

*AN1* patients with anorexia nervosa before therapy, *AN2* patients with anorexia nervosa after therapy, *HC* healthy controls

Mean and standard deviation presented

^*^p ≤ 05 versus AN2, ^**^p ≤ 0.01 versus AN2, ^***^p ≤ 0.001 versus AN2, ^#^ *p* ≤ 0.05 versus HC, ^##^ *p* ≤ 0,01 versus HC, ^###^ *p* ≤ 0.001 versus HC

### The effect of clinical and demographic variables on neurocognitive measures

Spearman correlations were performed between the mean accuracy scores and clinical variables (e.g. Yale-Brown and BDI, years of education, and current BMI.). In patients with anorexia, a statistically significant correlation was found between the mean score of enumeration RFFT and BDI (rho = 0.33; *p* ≤ 0.05) and enumeration task of RFFT and Y-BOCS-obsessive thoughts and Y-BOCS-compulsive behaviours (rho = 0.29; *p* ≤ 0.05 and rho = 0.28; *p* ≤ 0.01 respectively).

Interestingly, no statistically significant correlations were found in patients after therapy. In the control group, statistically significant correlations were found between the mean score of CWST-reading the names of colours in black (ms) and BMI (rho = − 0.26; *p* < 0.01) and the mean score of CWST-reading the names of colour in black (ms) and EAT-26 (rho = − 0.24; *p* < 0.01).

#### Evaluative survey results

The initial analysis has shown that participants evaluated the training as useful and motivating in taking up other activities of a therapeutic character. 90% of people thought that the objectives and the schedule of each session were realized to a large extent (the highest answer). Therapists’ competencies were also evaluated as high, especially as far as fulfilling patient expectations and the adapting participants’ possibilities to the training are concerned. Moreover, therapists were described as sensitive, emphatic, and outgoing. A tendency towards a critical evaluation of their own skills, which can correlate with a cognitive style being observed in people with anorexia nervosa, is noticeable (perfectionism, predominance of analytical thinking over synthetic thinking, intensified mental mistakes).

Answers to open-ended questions show that participants noticed benefits resulting from changes in the way of thinking, and the change of attitude to problem solving.

Below are presented the percentage results for the question about NT: ‘Neurocognition Training: participation in the training has helped you’: (Table 3).

**Table 3:** Results of patients’ satisfaction survey

Question/rating	A small extent	A moderate extent	A large extent
Improvement of concentration during everyday activities, e.g.: reading, conversation, learning	10%	50%	40%
Better orientation in the close and distant environment, improvement of visual and spatial perception, e.g.: when shopping, using maps, perception of visual illusions	10%	35%	55%
Improving my visual and auditory memory	10%	35%	55%
Development of abstract, logical, comprehensive thinking which is supportive in drawing conclusions and solving problems	10%	30%	60%
In setting goals, planning and carrying out daily duties	10%	35%	50%
Finding more creative solutions to everyday activities, creative thinking	10%	25%	65%
The development of cognitive flexibility by changing habits and ways of thinking	10%	50%	40%
Answer to the question how my cognitive processes operate, i.e. the so-called thinking about thinking; and how can it be developed?	10%	45%	40%
Discovery of a typical style of thinking, its advantages and disadvantages	10%	35%	55%

Additionally, statements by chosen participants about the benefits gained while participating in the session are given (Table 4).

**Table 4:** Benefits gained from participation in NT

Please mention what other benefits you have gained from participation in the Neurocognition Training (would you recommend NT to other people and for what reasons?)	
CHOSEN ANSWERS	
- ‘I’m not fussy about details’	
- ‘I have improved my concentration and I have started to think about my thoughts’	
- ‘I have learned to change the strategy when performing a task, I had a lot of fun during the classes and I fully recommend them to others’	
- ‘I have broadened the way of perceiving various situations’	
- ‘I have developed a skill to shift attention and concentration’	

## Discussion

The aim of the study was to assess the impact of the integrative model of the therapy including neurocognitive training on cognitive functioning of our anorexic adolescents.

The substantial body of research in this domain consistently suggests the presence of neurocognitive deficits in attentional switching, cognitive rigidity, and central coherence; [3, 14, 20, 22–24, 26, 28, 30, 44–53]. The findings of the current study corroborated those of the above -mentioned authors and stated that the adolescent patients in the acute phase of the illness reported some level of neurocognitive impairment on all examined measures while their test performances were significantly worse compared to healthy controls. However, as therapy progressed, statistically significant improvement was noted in patients who completed the hospital programme including the ten-week neurocognitive training.

Interestingly, the group of individuals with anorexia nervosa who successfully completed therapy performed similarly to healthy controls on the Ruff Figural Fluency Test (in total) and TMT B. These findings might suggest intact executive processes and attentional switching after the comprehensive therapeutic programme. It should be noted that regarding the use of the rotation strategy, the results of AN patients improved as a result of treatment, in contrast to the enumeration strategy and the error ratio, in which cases the analysis showed no differences between AN1 and AN2. Unfortunately, it turned out that the treatment does not significantly affect the error ratio, and the patients still scored higher in this regard than HC.

The deficit that persists after therapy was found in the speed of information processing regarding the performance of TMTA and CWST – reading the names of colours in black, where marked improvement was reported between patient groups**;** however, the healthy controls conducted tasks significantly faster compared to patients after therapy. These deficits appeared unrelated to BMI and clinical measures, with the exception of statistically significant correlations in the AN group before therapy between the enumeration task of RFFT and BDI (rho = 0.33; *p* ≤ 0.05) and the enumeration task of RFFT and Y-BOCS-obsessive thoughts and Y-BOCS-compulsive behaviours (rho = 0.29; *p* ≤ 0.05 and rho = 0.28; *p* ≤ 0.01 respectively).

Additionally, analyses showed statistically significant differences between patients and healthy controls in error rates of CWST for naming ink colours of incongruent words with more errors in patients after treatment**,** which again stayed in line with the results of other authors [54–59].

Apart from neurocognitive functioning, marked clinical improvement was observed in patients’ depression level, the severity of obsessions, and compulsions. These symptoms in the client group before discharge did not differ from those of controls.

With regards to core eating disorders symptoms per se*,* a remarkable improvement was reported in the patient group after therapy**,** although their symptoms intensity compared to healthy controls remained significantly higher.

A key challenge in ED research is to be addressed at examining the nature of existing neurocognitive deficits whether they are trait- or state-related disturbances. In fact, starvation, depression, and anxiety symptoms present in AN can contribute to these cognitive problems. However, these difficulties have been found to precede the onset of AN in childhood, and to persist to a less severe extent in recovered anorexia patients [60, 61] have proposed that these cognitive problems could be considered as maintaining factors for the disorder itself. This suggests that these problems may for some individuals represent underlying traits that preceded the onset of the disorder, rather than symptoms resulting from starvation [62].

We do support previous research affirming that neurocognitive deficits in anorexia could be a consequence of the illness but also a risk factor for it to develop.

The growing body of research consistently indicates structural brain changes in anorexia nervosa [2] which remain largely reversible in those patients achieving weight recovery [63, 64]. Both the hippocampi and anterior cingulate, crucial in processing memory, spatial reasoning, and set-shifting, are known as brain structures with a reduced thickness of grey matter in AN patients in the acute phase of the illness. Again, these brain regions were described as recovered in size throughout the treatment [65, 66].

Recently, cognitive remediation programmes have been developed to improve cognitive flexibility, central coherence, planning, and reasoning, potentially providing stimulation to these impaired brain regions in AN [67]. Further exploring of executive functioning deficits that seem to be characteristic of the illness has led to a new idea for an adjunctive treatment of AN in the form of cognitive remediation therapy [68]. This treatment aims to improve cognitive flexibility, in particular through repeated practice with skills such as set shifting, poor central coherence and attention to details [69]. At present, this treatment is being investigated for its potential to enhance efficacy with other evidence-based psychotherapy, such as cognitive behavioural therapy or the integrative model of psychotherapy commonly used in various clinical settings [41, 70, 71]. In our study, an integrative model of therapy including Neurocognitive Training appeared efficacious while all service users who underwent such treatment presented marked cognitive improvement.

The aim of this study was to assess the process of change in neurocognitive functioning throughout a comprehensive process of integrative psychotherapy including cognitive remediation to find out how CRT (Cognitive Remediation Therapy) enhances the efficacy of concurrent treatment in a cognitive domain. This appears important while in clinical inpatient setting integrative model of psychotherapy is being commonly delivered.

We acknowledged limitations within our study and will try to improve upon these shortcomings in future research and evaluation. First of all, it would be crucial to evaluate the efficacy of treatment as usual (TAU) in conjunction with CRT comparison to TAU with no CRT added.

The study was conducted using well-known batteries and scales and comprehensively structured neurocognitive training which was well received by service users.

Recruitment criteria in our study were highly selective**,** which made this group very homogeneous.

## Conclusion

To conclude, cognitive remediation programmes designed to train patients directly in the neurocognitive areas in which they are impaired may hold more promise. Our results, based upon well-established clinical and psychological measures, as well as patients’ survey, truly convinced us that neurocognitive training added to the hospital therapeutic programme and is a direction worth further research. However, further large-scale longitudinal investigations are needed in order to elaborate on the unclear relationships between neurocognition, symptoms and functional outcome.

## Additional files

Additional file 1: The dataset of the project. (XLS 63 kb)

## Abbreviations

AN: Anorexia nervosa
BDI: Beck depression inventory
BMI: Body mass index
CRT: Cognitive remediation therapy
CWST: Color-Word stroop task
EAT-26: Eating attitude test
HT: Healthy controls
NT: Neurocognitive training
RFFT: Ruff figural fluency test
TMT: Trail making test / TMT A and B
Y-BOCS: Yale-brown obsessive compulsive scale
